# Goal-setting intervention in patients with active asthma: protocol for a pilot cluster-randomised controlled trial

**DOI:** 10.1186/1745-6215-14-289

**Published:** 2013-09-11

**Authors:** Gaylor Hoskins, Purva Abhyankar, Anne D Taylor, Edward Duncan, Aziz Sheikh, Hilary Pinnock, Marjon van der Pol, Peter T Donnan, Brian Williams

**Affiliations:** 1 Nursing, Midwifery and Allied Health Professionals Research Unit, University of Stirling, Unit 13 Scion House, Stirling University Innovation Park, Stirling, Scotland FK9 4NF, UK; 2 Allergy & Respiratory Research Group, Centre for Population Health Sciences, The University of Edinburgh, Medical School Doorway 3, Teviot Place, Edinburgh, Scotland EH8 9AG, UK; 3 Health Economics Research Unit, University of Aberdeen, Foresterhill, Aberdeen, Scotland AB25 2ZD, UK; 4 Division of Population Health Sciences, Medical Research Institute, University of Dundee, The Mackenzie Building, Kirsty Semple Way, Dundee, Scotland DD2 4BF, UK

**Keywords:** Asthma, Goals, Behaviour change, Intervention, RCT, Primary care

## Abstract

**Background:**

Supporting self-management behaviours is recommended guidance for people with asthma. Preliminary work suggests that a brief, intensive, patient-centred intervention may be successful in supporting people with asthma to participate in life roles and activities they value. We seek to assess the feasibility of undertaking a cluster-randomised controlled trial (cRCT) of a brief, goal-setting intervention delivered in the context of an asthma review consultation.

**Methods/design:**

A two armed, single-blinded, multi-centre, cluster-randomised controlled feasibility trial will be conducted in UK primary care. Randomisation will take place at the practice level. We aim to recruit a total of 80 primary care patients with active asthma from at least eight practices across two health boards in Scotland (10 patients per practice resulting in ~40 in each arm). Patients in the intervention arm will be asked to complete a novel goal-setting tool immediately prior to an asthma review consultation. This will be used to underpin a focussed discussion about their goals during the asthma review. A tailored management plan will then be negotiated to facilitate achieving their prioritised goals. Patients in the control arm will receive a usual care guideline-based review of asthma. Data on quality of life, asthma control and patient confidence will be collected from both arms at baseline and 3 and 6 months post-intervention. Data on health services resource use will be collected from all patient records 6 months pre- and post-intervention. Semi-structured interviews will be carried out with healthcare staff and a purposive sample of patients to elicit their views and experiences of the trial. The outcomes of interest in this feasibility trial are the ability to recruit patients and healthcare staff, the optimal method of delivering the intervention within routine clinical practice, and acceptability and perceived utility of the intervention among patients and staff.

**Trial registration:**

ISRCTN18912042

## Background

Asthma affects more than 300 million people throughout the world with little evidence of a declining trend in prevalence [1,2]. In the UK, 5.2 million people are currently being treated for asthma and an estimated 1 in 7 of the population will be diagnosed with asthma at some stage in their lives [3]. Although the number of new cases of childhood asthma appears to be declining, the prevalence rate for adults continues to rise [4]. Asthma has a significant effect not only on an individual patient’s health-related quality of life (HRQoL) [5], but also on society and the economy through work absence and premature retirement [6,7]. Its impact on national health systems is considerable [4,8,9]. In the UK treatments for asthma and other allergic disorders account for more than 10% of all primary care prescribing costs [8]. Asthma management guidelines recommend that patients with asthma should be offered self-management support that focusses on individual needs and is reinforced by written personalised action plans [10]. The guidance is informed by growing evidence that personalised asthma management plans—incorporating a living with asthma plan, asthma medication plan and asthma action plan [11]—negotiated in the light of patient goals have the potential to improve a range of clinical outcomes and quality of life, thereby resulting in reduced drug bills, hospitalisations and costs to healthcare systems [10,12-14].

The emphasis on enabling self-management of asthma has led to a proliferation of asthma action plans being developed and recommended for routine use within primary as well as secondary asthma care [10]. Several studies have subsequently reported that asthma action plans tend to be under-promoted by health professionals and under-used by patients and carers, suggesting a gap between recommended and actual behaviour [15-19]. Further evidence suggested that important factors contributing to this gap are divergent perceptions of patients and professionals about asthma and its management and a mismatch between what patients want/need from these plans and what is provided by professionals [16,19]. The asthma action plans that are currently available are limited in scope, focussing predominantly on symptom management strategies (actions) to follow in the presence of deteriorating symptoms [14,20]. These medically focussed plans fail to incorporate patients’/carers’ views of asthma, its management within the context of their own lives and their personal asthma management strategies. As a result, many people with asthma fail to use them to help maximise their health potential, preferring to self-limit participation in activity to manage their symptoms [15,16]. To optimise the impact and use of asthma action plans, it has been suggested that they need to extend beyond the medical management of asthma to address patient goals in the wider context of their life and family and incorporate broader self-management strategies [19]. This is also consistent with current UK health policy, which reflects a paradigm shift away from the traditional, medical model of healthcare towards a patient-centred model of care that promotes collaborative partnership between patients and professionals in sharing information and agendas, setting goals and making decisions/plans for treatment and management [21,22].

The identification of goals is central to both the effectiveness and personalisation of self-management plans [23-25], in particular the likelihood that the plan will lead to changes in patients’ day-to-day self-management behaviours [24]. Goal-setting—a process in which people set themselves targets and work towards achieving these—is increasingly recognised as a potentially effective technique for assisting patients with chronic conditions to improve health-related behaviours and self-management [23-25]. The theory underlying health-related goal setting suggests that goals are more likely to be achieved when they are specific, important to patients, collaboratively set and there is a belief that these can be achieved. However, goal setting reflects only one of the processes involved in the self-management process [26]. Self-management, broadly defined as adoption and maintenance of health-improving behaviours, is conceptualised as comprising two different processes: motivational and self-regulatory [26,27]. The motivational process involves forming an intention to engage in a behaviour in order to achieve an outcome (goal) that is valued or a threat that is to be avoided, a belief that the behaviour will lead to desirable and valued outcomes, and a belief that s/he can engage in that behaviour [26]. The self-regulatory process involves translation of the behavioural intention into action, where ‘planning’ plays a useful role [28]. Planning consists of two further sub-processes serving different purposes [28]. The first involves specifying the intended action in terms of when, where and how to act—termed as action planning; the second involves anticipating obstacles to action and making specific plans to overcome them—termed coping planning. There is considerable evidence to suggest that action and coping planning increases the likelihood that patient behaviour will actually change (i.e. that intentions are translated into behaviour) [26-28]. This approach is supported by evidence showing the effectiveness of personalised asthma action plans when used in the context of regular reviews [29].

The theoretical underpinnings of self-management suggest that effective self-management support should involve interventions that target both the motivational as well as volitional phases of health behaviour change [26,27]. As providers of self-management support, health professionals must work with patients to identify goals (valued outcomes) that are important to patients, that may be achievable and with which they can engage as well as help patients make specific action and coping plans to achieve those goals. The identification of specific, personalised goals and associated feasible behaviours is a prerequisite for the creation of specific action and coping plans (i.e. forming an intention that spells out the when, where and how of goal striving in advance and planning coping strategies to deal with obstacles). Despite the central importance of these goals there is growing evidence to indicate that their identification in practice is far from easy [19,30]. There is a need therefore, to develop new, effective and practical approaches for eliciting goals and associated self-management behaviours by forming partnerships with patients that will support the self-management agenda [18]. The development of a simple goal-setting tool may boost this process by encouraging patients to focus their thinking about asthma and its effect on their life. This is an important step in prioritising management strategies with the patient. In this study, we test the feasibility and acceptability of using a goal-setting tool within a goal-setting intervention.

### Aims of the study

The primary aim is to pilot the design and process of a trial to test the effectiveness of a goal-setting intervention in the management of asthma in a primary care setting. We will assess our ability and refine approaches to recruiting practices, practice staff and patients to a definitive phase III randomised controlled trial (RCT), and establish the best way of delivering the intervention. Furthermore, we will conduct a value-of-information (VOI) analysis to inform the optimal sample size for any future trial [31].

The secondary aims are to estimate the impact of the goal-setting intervention on patient outcomes as well as the acceptability and perceived utility by both patients and health professionals of the goal-setting tool and the goal-setting process, and to inform the power of an economic evaluation in the full trial. The findings from this feasibility study will allow us to make final relevant modifications to the intervention prior to RCT development.

### Specific objectives

1. To pilot the intervention and its delivery in routine primary care asthma clinics.

2. To pilot the trial process including recruitment and performance of outcomes.

## Methods/design

### Trial design

We are conducting a pragmatic two-armed, multi-centre, single-blinded, clustered randomised controlled feasibility trial. Primary care practices will be randomly assigned to either an intervention or a control group. Patients in the intervention group practices will receive a goal-setting intervention within a ‘standard’ asthma review consultation. Patients in the control group practices will receive a ‘standard’ asthma review alone. Data will be collected from patients and patient records at baseline (before the intervention) and at follow-up of 3 and 6 months.

### Eligibility of practices for entering the trial

All general practices within two Scottish regional health boards will be invited to participate. Practices are eligible if they:

• have an asthma clinic run by a nurse in possession of an accredited asthma diploma who regularly reviews and manages patients with asthma.

• are willing to allow the practice nurse (PN) to attend a half-day training workshop.

### Eligibility of patients for entering the trial

All adults aged 18 years and over with a greater than 1-year history of asthma will be eligible to participate. Active asthma is defined as a diagnosis of asthma coded on the practice computer plus a prescription for an asthma medication in the previous year. Patients will be excluded if they have chronic obstructive pulmonary disease (COPD) or any other significant lung disease; are unable to give consent because of major medical, social or communication reasons; or are taking part in any other clinical trials involving the management of asthma.

### Allocation of trial interventions

The general practice is the unit of allocation. Central randomisation by the Tayside Clinical Trials Unit (TCTU) to intervention or control arms will be carried out separately for each of the two participating health board areas. This will be done using minimisation based on achieving optimum balance for practice list size (two strata <6,000 and >6,001 patients currently registered). The reason for randomisation by area is to ensure an even distribution of intervention and control practices as there is potential for geographical variation in asthma management strategies between health boards. Thus, within each area two large and two smaller practices will be matched to receive the intervention and control. TCTU will tell the research team of the outcome of the randomisation process and in turn the practices will be informed of their allocated group.

### Recruitment

#### General practices and healthcare professionals

The Scottish Primary Care Research Network (SPCRN) will assist with practice recruitment by writing to the practices informing them of the study and enclosing an information sheet, an expression of interest form and a project practice consent form. Additional informal contact in the form of a project flyer will be made with practice nurses via the National Health Service (NHS) Education for Scotland (NES) education facilitators for the two regions. The Respiratory Managed Clinical Networks (MCNs) in both areas will be asked to bring the project to the attention of the practices. Interested practices will be asked to return the expression of interest form to the SPCRN administrator. A reminder letter will be sent by the SPCRN 4 weeks after the initial postal date with one follow-up phone call 2 weeks later. Active recruitment will cease once at least eight practices (four in each arm) meeting the eligibility criteria have been recruited. Once recruited, arrangements will be made with the practice manager and practice nurse to complete the signing of the consent form and to discuss the trial protocol, patient recruitment and arrangements for pre-study training. A folder containing the core trial documents will be given to each participating practice. This will include an algorithm for selecting patients, patient information and consent forms, the research team’s contact details and all trial information.

#### Patient recruitment

Using the electronic asthma review recall reminder procedures, practices will be asked to identify all eligible patients aged 18 years plus due an asthma review within the proceeding 3-month period. The practice will then write to potential participants informing them that they are due an asthma review and that the practice is participating in the asthma goal project. A template of a covering letter—stating the study arm to which the practice has been allocated—has been provided for this purpose although practices can opt to create/use their own letter. Patients being aware of the study procedure before consenting to take part could lead to a difference in the type of patients participating in the intervention and in the usual care group. Any differences will be identified and controlled for in the final analysis. Accompanying this covering letter will be a project participant information sheet, a consent form and a short screening questionnaire. Interested patients will complete and return the consent form and the screening questionnaire in a pre-paid envelope to the Nursing, Midwifery and Allied Health Professionals research unit (NMHAP-RU) prior to arranging and attending for their asthma review. Patients will be asked to express on the screening questionnaire their preferred day and time to receive a telephone call. All recruitment materials have been approved by East of Scotland Research Ethics Committee (REC 2).

Eligible participating patients will be assigned a unique study identifier and their personal contact details and the original signed consent form scanned and electronically stored in a password-protected folder. One copy of the consent form will be sent to the practice to be inserted into the trial folder along with a letter informing the GP/nurse that the patient had consented to participate in the trial and one copy will be returned to the patient. The original paper copies will be destroyed in accordance with data protection guidelines. A thank-you letter will be sent to all patients who have indicated interest but are not considered eligible to participate informing them that their contact details and consent form will be destroyed safely and in accordance with data protection guidelines.

### The goal-setting intervention

The intervention consists of two components—one operating at patient level and the other at healthcare professional (HCP) level. At the patient level, the intervention consists of completion of a goal-setting tool (Additional file 1) designed to help them clarify goals in the context of their life and family as well as goals related to the management of their asthma; a ‘standard’ review of asthma during which a discussion will take place focussed on the patient’s goals and priorities; construction of a negotiated, tailored action plan (Additional file 2). At the level of HCP, it consists of training in developing shared goals and action plans with patients to facilitate achievement of the prioritised goals. The two components are described in detail below. The intervention draws on the findings from earlier work [19,30,32,33] as well as established theoretical concepts and empirical evidence relevant to each specific step in the intervention process [23-26,28]. This integration of theory with findings from the primary empirical study strengthens the basis of the intervention and is supported by recommendations in the Medical Research Council (MRC) Framework for Complex Interventions [34]. The control group receives a ‘standard’ review of asthma only.

#### Goal-setting tool

The goal-setting tool consists of three sections. It begins by explaining the meaning and importance of goal setting, the purpose of the tool and instructions on completing the tool. Section one aims to elicit patients’ goals in day-to-day life by asking them to write down what they would really like to do or achieve, list them in order of priority and indicate whether asthma makes it difficult to achieve them. Section two aims to establish whether the goals were important for their own sake (an end in themselves) or important because they help patients achieve something else (transitionary). This is done by asking patients to think about how achieving the goals would benefit them. Finally section three aims to elicit goals specific to asthma management, their perceived importance and patients’ confidence in achieving them. Each section includes a completed example for illustrative purposes.

#### Healthcare professional training

Practices allocated to the intervention arm will be asked to name the practice nurse who will be participating in the study. All nominated nurses will attend a workshop where they will receive training on trial procedure, the components of a ‘standard’ asthma review and the use of goal setting in the management of asthma. Specifically, the nurses will be trained in using the information from the goal-setting tool to focus a discussion with patients around their life goals and priorities, integrating patient life goals with asthma management goals, negotiating and prioritising shared goals, and developing specific action plans to facilitate the achievement of those goals. The workshop will be delivered by the project research fellows and project PI. The programme for this course is available as Additional file 3. The nominated nurses in practices randomised to the control arm will attend the first session of the workshop relating to project procedure and asthma review only.

#### Personalised action plans

To facilitate the achievement of prioritised goals, the patient, in negotiation with the nurse, will create personalised action and coping plans. For each goal, they are asked to identify up to three steps towards the goal, specify the detailed plan for each step, anticipate the obstacles to carrying out the plan and come up with plans to overcome the obstacles. Following the development of action plans, the patients are asked to decide whether they would like a follow-up visit with the nurse, when and whether in-person or via telephone, to discuss their progress with implementing the action plans. Follow-up based on clinical need will be at the discretion of the nurse.

### Trial procedures

Once a patient has consented to take part in the trial by returning the expression of interest form and signed consent, they will be telephoned by a researcher, at which time their eligibility to participate in the project will be confirmed and the baseline questionnaires completed. Patients will then be asked to contact their practice and arrange an appointment for an asthma review. The research team will liaise with the general practice to ensure all consenting patients who have completed baseline questionnaires have made an appointment. Patients will be seen by the PN in their usual clinic setting.

Patients in the intervention group practices will be sent the goal-setting tool prior to the review appointment with instructions to complete it independently at their convenience and to take it with them to the review appointment. Patients not bringing it to the review will be asked to complete it in the waiting area prior to the review.

The nurse in both study groups will conduct a review consultation in line with clinical guidelines [control assessment; peak expiratory flow (PEF); check of inhaler technique; review of medication, etc.] In addition the nurse in the intervention group will review the patient’s own goals as identified using the goal-setting tool. Nurse and patient will discuss the individual goals and, if necessary, the appropriateness of and the priority given to each goal. An individualised action plan will be discussed, negotiated and agreed upon, tailored to the number and complexity of the elicited goals. The written record of the personalised GOAL action plan will be given to the patient. This plan will be in addition to any symptom-related asthma action plan provided by the practice. Nurses in both the intervention as well as control groups will be asked to audio-record the review consultations using a digital recorder provided by the research team.

Patients receiving the intervention will be contacted by the researcher, via the telephone, 6 weeks after their review consultation to elicit their perceived progress in implementing their plans and perceived efficacy of the plans in achieving associated goals. Follow-up outcome questionnaires will be completed at 3 months and again at 6 months post review for all patients. Patients will have the option of receiving these by post and completing it themselves, or to complete them via the telephone with a researcher. The former will be supported by a telephone reminder to patients where necessary.

### Measures and measurement instruments

• Asthma-related quality of life: Assessed using the mAQLQ [35]. The mAQLQ measures the functional problems experienced by people with asthma. This is a validated and widely used tool in clinical trials [36,37] designed to ask adults with asthma about the physical, emotional occupational, and social problems most troublesome to them on a day-to-day basis. A clinically relevant improvement is an increase of ≥0.5 in individual mAQLQ mean scores. We will also compare mAQLQ scores at 3-months post-randomisation to establish the most appropriate time-point for assessment of clinical endpoints.

• Asthma Control Questionnaire (ACQ) [38]: The shortened version of the ACQ has six questions (the top scoring 5 symptoms and daily rescue bronchodilator use). Patients are asked to recall how their asthma has been during the previous week and to respond to the symptom and bronchodilator use questions on a 7-point scale (0 = no impairment, 6 = maximum impairment). The questions are equally weighted and the ACQ score is the mean of the six questions and therefore between 0 (totally controlled) and 6 (severely uncontrolled).

• Healthcare resource use (HSRU): This is a reflection of asthma control. Data will be collected from patient records using a form designed for use in previous studies [39,40]. Data will include the number and type of asthma-related consultations, number of exacerbations, emergency asthma medication use, etc., in the 6 months pre- and post-intervention.

• Perception of patient empowerment using the Patient Enablement Instrument [41]: This six-question (4 responses) tool measures a patient’s confidence, understanding and ability to cope with their health and illness.

• Quality of life using the EQ-5D-3 L [42]: Applicable to a wide range of conditions, this simple questionnaire is quick to complete. It measures health outcomes by classifying patients into one of 243 health states (5 dimensions, each with 3 levels). The five dimensions are mobility, self-care, usual activities, pain/discomfort and anxiety/depression. The data are translated into ‘utility scores’ using the UK population tariff (Dolan, 1995) and used to estimate Quality Adjusted Life Years in cost-effectiveness analysis.

### Outcome measures

The proposed outcome measures for the definitive trial have been chosen to reflect both clinical and patient-centred factors. The main primary outcome measure will be change in quality of life as measured by the mini Asthma-related Quality of Life Questionnaire (mAQLQ) [35]. The proposed secondary outcomes will include measures of asthma control, healthcare resource use, patient self-efficacy and cost-effectiveness. Asthma control will be assessed from the scores on the Asthma Control Questionnaire (ACQ) [38] and data from the Health Services Resource Use (HSRU) questionnaire. Cost-effectiveness will be measured as a cost per Quality-Adjusted Life Year (QALY) of the intervention compared to the control group. Utilising the HSRU data, the costs of overall asthma-related healthcare resource use over the 6-month pre- and post-trial period from an NHS perspective will be calculated. QALYs over the 6-month trial period will be derived from EQ-5D-3L responses [43]. The GOAL-setting process will be assessed by recording the number and type of goals identified and achieved. This information will be gleaned from the patient-completed goal setting tool and their level of confidence for achieving their identified goals from their completed action plan.

For this pilot trial, the key outcome measures of interest are aimed at enabling us to assess the feasibility of and plans for conducting the definitive trial:

• Practice and patient recruitment rates

• Retention rates

• Likely cost of the intervention

• Acceptability of GOAL tool and action plan process to adults with asthma

• The variability of the main outcome measure

• The sample size required to effect change in asthma-related quality of life

• Acceptability, effectiveness and ease of use of the chosen measuring instruments

### Sample size calculation

As this is a feasibility study we have not undertaken a formal sample size calculation. A sample size of eight practices will allow for four practices in each health region. The aim will be to recruit approximately 80 patients in total (40 in each arm).

### Fidelity: practice and practice nurse

Commitment to the project process will be facilitated by the use of clear written information on what the study involves; support for recruitment by the SPCRN; use of individual patient case report forms to log each stage of the project process; guidance to practices on identifying eligible patients; a clear account of payment for practice time; and an extensive training workshop for participating nurses. The project researchers will be in regular contact with the practices to offer support, answer any questions and solve any problems.

### Withdrawal of patients from the study

There are five points at which consenting patients could withdraw from the study:

(1) prior to completing baseline questionnaires

(2) prior to attending the asthma review appointment with the practice nurse

(3) prior to the 6-week telephone follow-up

(4) prior to completing the 3-month follow-up questionnaires

(5) prior to completing the 6-month follow-up questionnaires

### Data management

The TCTU will be responsible for data management, data quality assurance, backup, business recovery and statistical analysis.

### Statistical analysis

Data analysis, using the following analysis plan, will be undertaken blind to the allocation arm. The primary analysis will be a per protocol analysis based on an intention to treat [44].

### Descriptive statistics

#### Describing baseline characteristics of patients and practices

For each study arm, we will describe:

Baseline characteristics of patients: age (mean and SD), gender (number and percentage), quality of life and asthma control.

Baseline characteristics of practices: practice list size (median and IQR, or mean and SD if normally distributed), number of patients on the practice list with an asthma code and the number of patients with active asthma (median and IQR, or mean and SD if normally distributed), age distribution (number and percentage), and deprivation and rurality [proxy measures based on the Information Services Division (ISD) Scotland classification] [45].

#### Comparison between treatment arms

The difference in the validated mAQLQ score between the intervention and control groups at baseline and 3 and 6 months post-intervention will be compared, adjusting for practice level stratum (region and list size). A clinically relevant improvement is an increase of ≥0.5 in individual mAQLQ mean scores [35]. Quality of life using the validated mAQLQ was measured at the baseline (January to May 2013) prior to the HCP asthma review appointment, and repeated at 3 months and 6 months post-intervention. Comparison of the mAQLQ scores at 3 months post-intervention as well as at 6 months post-intervention will enable us to establish the most appropriate time point for assessment of clinical endpoints. Changes from baseline to six months post intervention will be assessed in SPSS for Windows using multiple regression analysis with 6-month mAQLQ as outcome and baseline mAQLQ as covariate along with arm of trial (intervention/control) in the model. We will be adjusting all models for clustering within practices and matching by size. Transformations of the outcome variables will be used where necessary if these are not normally distributed. All analyses will be stratified by the stratification factors. We will estimate an ICC for all potential outcomes in the full trial. Consequently sample size estimates will be made for the full trial.

#### Cost-effectiveness

Cost-effectiveness of the asthma goal-setting tool will be measured as a cost per QALY [43]. The incremental cost-effectiveness ratio (ICER) will be estimated by dividing the difference in mean total costs between the intervention and control group by the difference in QALYs. Bootstrapping will be used to get an estimate of variability in the ICER. The economic analysis will include a VOI analysis [31]. The results of this analysis will indicate whether the data provided by the phase II feasibility trial are sufficient for decision-making and, if it is not sufficient, the optimal sample size of a future trial needed to provide additional information. The VOI analysis will be based on the estimates of the mean, variance and covariance of differences in costs and QALYs obtained from this phase II feasibility trial.

### Missing data

#### mAQLQs

The mAQLQ is divided into four domains, with varying numbers of questions per domain, and the overall mAQLQ score is calculated from the mean of each domain [35]. Where more than one response is missing in the symptom or activity domains and any response missing in the emotional function or environmental domains, this patient will be excluded from the analysis [46].

### Reporting and dissemination

Reporting will adhere to revised CONSORT criteria for cluster RCTs [47].

### Qualitative phase

Qualitative research methods will address three key questions: how the goal-setting tool was received, how the elicited information was used, and the effect on work practice and the self-management process.

### Qualitative interviews

As each practice achieves their recruitment target, practice nurses in both arms of the trial will be invited to take part in a qualitative interview. In addition, a purposive sample of patients will be asked to participate in an interview. The patient interviews will be carried out after the 3-month follow-up data have been collected. We hypothesise that the nature of goals and the subsequent experience of engaging in the goal-setting process is likely to be influenced by a patient’s gender, age, asthma severity, deprivation, educational status and the practice nurse. Therefore, we purposively aim to select patients from both the genders, from varying age groups, with varying levels of asthma severity and seeing different nurses. We are planning for approximately 20 interviews—all of the intervention practice nurses and at least 10 patients. We believe that with this number we are likely to achieve data saturation on the questions of interest.

Interviews will be semi-structured and guided by a patient or professional focussed interview schedule. Patient interviews will focus on four key topics.

• reasons for and experiences of taking part in the trial

• acceptability and perceived usefulness of the goal-setting tool

• experience of and perceived value of the goal-setting process

• perceived change in the communication with HCPs

• perceived impact on asthma management and quality of life.

HCP interviews will focus on the following topics:

• experiences and acceptability of all elements of the trial

• experiences of using the information from the goal-setting tool and facilitating the development of personal management plans

• perceived change in communication with patients

• perceived impact on clinical practice

Patients recruited to the study will already have consented to be part of the interview process. A purposive sample of patients will be contacted by telephone to confirm they wish to go ahead with the interview. If they agree an interview time will be arranged. Prior to this interview a second consent form will be completed. Interviews will be conducted in a confidential, private setting, e.g., the patient’s home or the health professional’s place of work. Telephone interviews will be considered if face-to-face interviews prove difficult to arrange. Interviews will be conducted by the same interviewer and are expected to last an average of 30–45 min. All interviews will be audio-recorded, transcribed verbatim and checked for accuracy. The interview transcripts will be returned to the interviewees for verification.

### Data management and analysis

All interview transcripts will be given a project code at the point of collection. Any identifiable data will be changed to ensure anonymity for participants. All the qualitative data will be managed using the data management software NVivo 9. Data will be analysed following the guidelines for thematic framework analysis [48], which allows systematic classification and organisation of the data in terms of key themes and emergent patterns. The analytical process will be an iterative one with new issues identified during interim data analysis included in subsequent interviews. An initial coding frame will be developed using data from the first few transcripts and key concepts from the psychological theories underlying the intervention. The coding frame will then be applied systematically to all the transcripts, adding new themes and categories as they emerge from the data. Once all the text has been coded, the coding frame will be refined by revisiting and sorting the text in each category, searching for association among different themes and grouping them under more comprehensive, higher order themes. Various strategies will be employed throughout the data analysis to ensure trustworthiness of the data: content checking of the transcripts by the interviewees, data coding and checking for accuracy by two researchers, constantly searching for alternative explanations, and discussion of emerging themes and patterns with the wider research and project management team. The findings from the qualitative research will be merged with the findings from the quantitative data and used to design the full RCT.

### Recordings of asthma reviews

All consultations, whether in the intervention or control arm, will be recorded and listened to independently. This will allow us to check the fidelity of the review process.

### Trial steering committee

The Feasibility Trial Steering Committee (FTSC) will monitor and supervise the trial and comment on any proposed amendments to the protocol. The FTSC is chaired by Dr Gaylor Hoskins. Professor Brian Williams, Professor Aziz Sheikh, Professor Peter Donnan, Dr Hilary Pinnock, and Dr Marjon van der Pol are study co-applicants and sit on the FTSC. Dr Purva Abhyankar and Dr Anne Taylor, the research managers for the study, also sit on this group. Mr Gordon Brown (Asthma UK Scotland) and Mrs Wendy Nganasurian represent the patient perspective. Professor Chris Griffiths is acting as an independent monitor for the study. The FTSC has agreed to operate within the framework suggested in the MRC Guidelines for good clinical practice in clinical trials [49].

### Ethical considerations

The clinical trial will be conducted according to the Helsinki Declaration [50], Good Clinical Practice guidelines [49] and NHS research governance requirements. Patients who have agreed to allow the study team to access their clinical records have provided written informed consent. All patients were made aware that they could withdraw from the research at any time. The study has been approved by the East of Scotland Research Ethics Committee (REC ref. no. 12/ES/0050). All appropriate NHS Research and Development approvals have been obtained.

### Study timeline

• Trial start: 1 October 2012

• Baseline data collection: From January 2013 until all patients have been recruited

• Interventions in general practice: January 2013 (training); January 2013 onwards (patient recruitment), February 2013 onwards (patient review appointments with PNs)

• End of interventions and follow-up in general practice: November 2013

• Qualitative interviews: July to November 2013

• Start of data analysis: November 2013

• Planned study end date: end December 2013

• Duration: 15 months

## Trial status

At the time of submission ten practices are participating in the trial and patients are currently being recruited.

## Abbreviations

RCT: Randomised controlled trial; HRQoL: Health-related quality of life; SD: Standard deviation; ICC: Intra-cluster correlation; VOI: Value of information; BTS: British Thoracic Society; COPD: Chronic obstructive pulmonary disease; SPCRN: Scottish Primary Care Research Network; NHS: National Health Service; NES: NHS Education for Scotland; MCNs: Managed clinical networks; GP: General practitioner; PI: Principal investigator; NMAHP-RU: Nursing, Midwifery and Allied Health Professionals Research Unit; HCP: Healthcare professional; MRC: Medical research council; PN: Practice nurse; PEFR: Peak expiratory flow rate; TCTU: Tayside clinical trials unit; mAQLQ: Mini asthma quality of life questionnaire; ACQ: Asthma control questionnaire; HSRU: Health services research unit; QALY: Quality-adjusted life years; IQR: Interquartile range; SPSS: Statistical package for Social Sciences; ICER: Incremental cost-effectiveness ratio; PMG: Project management group; FTSC: Feasibility trial steering committee; ISD: Information services division.

## Competing interests

The authors declare that they have no competing interests.

## Authors’ contributions

All authors have contributed to the design of the study and the preparation of the draft manuscript. GH, as chief investigator, co-conceived the study, wrote the study protocol, designed the study materials, applied for ethics and NHS R&D approvals, and drafted the manuscript; PA and AT have co-managed the study, collected the patient outcomes, conducted the patient and health professional interviews, and contributed to the manuscript. ED, a grant holder, contributed to the design and coordination of the study, particularly the conceptualisation of the theory upon which the intervention is based, and commented on the draft manuscript. AS and HP contributed to designing the study, are grant holders and commented on the draft manuscript. MP, a grant holder, provided health economics input for both the study protocol and the draft manuscript. PD, a grant holder, provided the statistical analysis framework for the study and commented on the draft manuscript. BW co-conceived the study, participated in its design and coordination, and commented on the draft manuscript. All authors read, commented on and approved the final manuscript.

## Authors’ information

GH is a clinical researcher with a background in primary care respiratory management and asthma research. BW is a social scientist with an extensive portfolio in qualitative research in public health and primary care who has conducted a number of asthma-related projects and has experience in general practice behaviour and guideline interpretation and implementation; PD is a bio-statistician with experience in asthma-related research and randomised control trial techniques; MvdP is a health economist with an extensive portfolio of research into the cost benefits of health interventions; AS is an epidemiologist and professor of general practice with expertise in the design and implementation of cluster and parallel groups RCTs; HP is a general practitioner with expertise in asthma who sits on the BTS/SIGN asthma guideline steering group, and has experience in development and evaluation of complex interventions; ED is an occupational therapist and Senior Research Fellow in the NMAHP-RU with a portfolio of work that includes goal-related issues. PA is a psychologist with experience in conducting and analysing qualitative research; AT is a nurse and clinical research fellow. The principal investigator (GH) has been a recipient of the CSO Primary Care Research Award and currently holds a clinical research fellowship with the CSO-funded NMAHP Research Unit at the University of Stirling. HP is currently a recipient of the CSO Primary Care Research Award. BW is the director and ED a senior researcher with the Nursing, Midwifery & Allied Health Professional (NMAHP) Research Unit.

## Supplementary Material

Additional file 1 Goal-setting tool. Click here for file

Additional file 2 Goal action plan. Click here for file

Additional file 3 Practice nurse training schedule. Click here for file

## References

[B1] Masoli M Fabian D Holt S Beasley R The global burden of asthma: executive summary of the GINA Dissemination Committee report Allergy 2004 59 469 478 10.1111/j.1398-9995.2004.00526.x 15080825

[B2] Anandan C Nurmatov U van Schayck OCP Sheikh A Is the prevalence of asthma declining? Systematic review of epidemiological studies Allergy 2010 65 152 167 10.1111/j.1398-9995.2009.02244.x 19912154

[B3] Asthma UK Where Do We Stand 2004 http://www.asmabronquica.com.br/medical/wheredowestand%5B1%5D.pdf?id=92

[B4] Simpson CR Sheikh A Trends in the epidemiology of asthma in England: a national study of 333,294 patients J R Soc Med 2010 103 3 98 106 10.1258/jrsm.2009.090348 20200181 PMC3072257

[B5] Asthma UK Living on a knife edge 2004 53 88 http://www.nice.org.uk/nicemedia/live/13550/61417/61417.pdf

[B6] Weiss KB Sullivan SD The health economics of asthma and rhinitis. I. Assessing the economic impact J Allergy Clin Immunol 2001 107 3 8 10.1067/mai.2001.112262 11149982

[B7] Yelin E Katz P Balmes J Trupin L Earnest G Eisner M Blanc P Work life of persons with asthma, rhinitis, and COPD: a study using a national, population-based sample J Occup Med Toxicol 2006 1 2 10.1186/1745-6673-1-2 16722563 PMC1436006

[B8] Gupta R Sheikh A Strachan DP Anderson HR Burden of allergic disease in the UK: secondary analyses of national databases Clin Exp Allergy 2004 34 520 526 10.1111/j.1365-2222.2004.1935.x 15080802

[B9] Anandan C Gupta R Simpson CR Fischbacher C Sheikh A Epidemiology and disease burden from allergic disease in Scotland: analyses of national databases J R Soc Med 2009 102 431 442 10.1258/jrsm.2009.090027 19797601 PMC2755331

[B10] British Thoracic Society & the Scottish Intercollegiate Guidelines Network British guideline on the management of asthma Guideline No. 101 2012 http://www.sign.ac.uk/guidelines/fulltext/101/index.html

[B11] Ring N Pinnock H Wilson C Hoskins G Jepson R Wyke S Sheikh A Asthma plans: Understanding what we mean. Linguistic analysis of terminology as used in published texts Prim Care Respir J 2011 20 2 170 177 10.4104/pcrj.2011.00012 21445536 PMC6549815

[B12] Gordon C Galloway T Review of Findings on Chronic Disease Self-Management Program (CDSMP) Outcomes: Physical, Emotional & Health-Related Quality of Life, Healthcare Utilization and Costs Centers for Disease Control and Prevenention and National Council on Aging 2008

[B13] Kennedy A Reeves D Bower P Lee V Middleton E Richardson G Gardner C Gately C Rogers A The effectiveness and cost effectiveness of a national lay-led self care support programme for patients with long-term conditions: a pragmatic randomised controlled trial J Epidemiol Community Health 2007 61 3 254 261 10.1136/jech.2006.053538 17325405 PMC2652924

[B14] Lahdensuo A Guided self management of asthma-how to do it BMJ 1999 319 759 760 10.1136/bmj.319.7212.759 10488007 PMC1116599

[B15] Brown R Behavioural issues in asthma management Pediatr Pulmonol 2001 32 Suppl21 26 30 11475168

[B16] Jones A Pill R Adams S Qualitative study of views of health professionals and patients on guided self-management plans for asthma BMJ 2000 321 1507 1510 10.1136/bmj.321.7275.1507 11118179 PMC27554

[B17] Hoskins G McCowan C Donnan P Friend J Osman L Results of a national asthma campaign survey of primary care in Scotland International J Qual Health Care 2005 17 3 209 215 10.1093/intqhc/mzi036 15831548

[B18] Pinnock H Thomas M Tsiligianna I The international primary care respiratory group (IPCRG) research needs statement 2010 Prim Care Resp J 2010 19 1 S1 S20 10.4104/pcrj.2010.00021 PMC6602279 20514388

[B19] Ring N Jepson R Pinnock H Wilson C Hoskins G Sheikh A Wyke S Encouraging the promotion and use of asthma action plans: a cross study synthesis of qualitative and quantitative evidence Trials 2012 13 216 doi:10.1186/1745-6215-13-216. http://www.trialsjournal.com/content/13/1/216 10.1186/1745-6215-13-216 PMC3561124 23164151

[B20] Zemek R Bhogal SK Ducharme FM Systematic review of randomized controlled trials examining written action plans in children. What is the plan? Arch Pediatr Adolesc Med 2008 162 2 157 163 10.1001/archpediatrics.2007.34 18250241

[B21] Scottish Executive Health Department Better Health, Better Care: A Discussion Document 2007 http://www.scotland.gov.uk/Publications/2007/08/13165824/0

[B22] Department of Health Healthy lives, Healthy People: Our strategy for public health in England 2010 http://www.official-documents.gov.uk/document/cm79/7985/7985.asp

[B23] Bandura A Self-efficacy: toward a unifying theory of behavioural change Psychol Rev 1977 84 191 215 847061 10.1037//0033-295x.84.2.191

[B24] Hardeman W Johnston M Johnston DW Bonetti D Wareham NJ Kinmonth AL Application of the theory of planned behaviour change interventions: a systematic review Psychol Health 2002 17 123 158 10.1080/08870440290013644a

[B25] Floyd D Prentice-Dunn S A meta-analysis of research on protection motivation theory J of Applied Sol Psych 2000 30 2 407 429 10.1111/j.1559-1816.2000.tb02323.x

[B26] Gollwitzer PM Sheeran P Implementation intentions and goal achievement: a meta-analysis of effects and processes Adv Exp Soc Psychol 2006 38 69 119

[B27] Schwarzer R Modeling health behavior change: how to predict and modify the adoption and maintenance of health behaviors Appl Psychol 2008 57 1 1 29 10.1111/j.1464-0597.2007.00325.x

[B28] Sniehotta F Towards a theory of intentional behaviour change: plans, planning, and self-regulation Br J Health Psychol 2009 14 2 261 273 10.1348/135910708X389042 19102817

[B29] Gibson PG Powell H Wilson A Abramson MJ Haywood P Bauman A Hensley MJ Walters EH Roberts JJL Self-management education and regular practitioner review for adults with asthma (Review) Cochrane Libr 2009 Issue 3 The Cochrane Collaboration http://www.thecochranelibrary.com 10.1002/14651858.CD001117 12535399

[B30] Williams B Steven K Sullivan F Tacit and transitionary: an exploration of patients’ and primary care health professionals’ goals in relation to asthma Soc Sci Med 2011 72 8 1359 1366 10.1016/j.socscimed.2011.02.038 21458126

[B31] Willan A Pinto EM The expected value of information and optimal clinical trial design Stat Med 2005 24 1791 1806 10.1002/sim.2069 15806619

[B32] Ring N Malcolm C Wyke S MacGillivray S Dixon D Hoskins G Pinnock H Sheikh A Promoting the use of personal asthma action plans: a systematic review Prim Care Respir J 2007 16 5 271 283 17710351 10.3132/pcrj.2007.00049 PMC6634230

[B33] Ring N Jepson R Hoskins G Wilson C Pinnock H Sheikh A Wyke S Understanding what helps or hinders asthma action plan use: a systematic review and synthesis of the qualitative literature Patient Educ Couns Online publication 2011 doi:10.1016/j.pec.2011.01.025 10.1016/j.pec.2011.01.025 21396793

[B34] Medical Research Council Developing and evaluating complex interventions 2010 http://www.mrc.ac.uk/complexinterventionsguidance

[B35] Juniper EF Guyatt GH Cox FM Ferrie PJ King DR Development and validation of the mini asthma quality of life questionnaire Eur Respir J 1999 14 32 38 10.1034/j.1399-3003.1999.14a08.x 10489826

[B36] Pinnock H Bawden R Proctor S Wolfe S Scullion J Price D Sheikh A Accessibility, acceptability, and effectiveness in primary care of routine telephone review of asthma: pragmatic, randomised controlled trial BMJ 2003 326 477 479 10.1136/bmj.326.7387.477 12609944 PMC150181

[B37] Ross J Yang Y Song PXK Clark NM Baptist AP Quality of life, health care utilization, and control in older adults with asthma J Allergy & Clin Immunol: In Practice 2013 1 2 157 162 10.1016/j.jaip.2012.12.003 24565454

[B38] Juniper EF Svensson K Mork AC Stahl E Measurement properties and interpretation of three shortened versions of the asthma control questionnaire Respir Med 2005 99 5 553 558 10.1016/j.rmed.2004.10.008 15823451

[B39] Pinnock H McKenzie L Price D Sheikh A Cost-effectiveness of telephone or surgery asthma reviews: economic analysis of a randomised controlled trial Br J Gen Pract 2005 55 119 124 15720933 PMC1463186

[B40] Ryan D Pinnock H Lee AJ Tarassenko L Pagliari C Sheikh A Price D The CYMPLA trial. Mobile phone-based structured intervention to achieve asthma control in patients with uncontrolled persistent asthma: a pragmatic randomised controlled trial Prim Care Respir J 2009 18 4 343 345 10.4104/pcrj.2009.00064 19940961 PMC6619373

[B41] Howie JRG Heaney D Maxwell M Walker JJ A comparison of a Patient Enablement Instrument (PEI) against two established satisfaction scales as an outcome measurement in primary care consultations Fam Pract 1998 15 165 171 10.1093/fampra/15.2.165 9613486

[B42] Rabin R de Charro F EQ-5D: a measure of health status from the EuroQol Group Ann Med 2001 33 337 343 10.3109/07853890109002087 11491192

[B43] Brooks R EuroQol: the current state of play Health Policy 1996 37 1 53 72 10.1016/0168-8510(96)00822-6 10158943

[B44] Hollis S Campbell F What is meant by intention to treat analysis? Survey of published randomised controlled trials BMJ 1999 319 670 674 10.1136/bmj.319.7211.670 10480822 PMC28218

[B45] Information Services Division Scotland General Practice - GP workforce and practice population statistics to 2012 http://www.isdscotland.org/Health-Topics/General-Practice/Publications/data-tables.asp?id=618#618

[B46] Juniper E Asthma Quality of Life Questionnaires (AQLQ(S), Mini AQLQ and Acute AQLQ) 1991 QOL Technologies Ltd

[B47] Campbell M Elbourne DR Altman DG CONSORT Group CONSORT statement: extension to cluster randomised trials BMJ 2004 328 7441 702 708 10.1136/bmj.328.7441.702 15031246 PMC381234

[B48] Ritchie J Spencer L Bryman A, Burgess RG Qualitative data analysis for applied policy research Analysing Qualitative Data 1994 London: Routledge

[B49] Medical Research Council MRC Guidelines for good clinical practice in clinical trials http://www.mrc.ac.uk/Utilities/Documentrecord/index.htm?d=MRC002416

[B50] World Medical Association World Medical Association Declaration of Helsinkihttp://www.wma.net/en/30publications/10policies/b3/17c.pdf

