# Supplementary material for: Goal-setting intervention in patients with active asthma: protocol for a pilot cluster-randomised controlled trial
Source: Trials. 2013 Sep 11;14:289. doi: 10.1186/1745-6215-14-289 (PMC3846716; doi:10.1186/1745-6215-14-289)
Supplement: Additional file 1 — Goal-setting tool. [file 1745-6215-14-289-S1.docx]

**Additional file 1: Goal Setting Tool**

Your Goals

Many people have goals or aspirations in life — these may be the things we want to accomplish, things that are important to us or things that we want from life.

Some of these goals may be quite clear to us as we think about them often (e.g. going on holiday in a few months time). However, other goals can be less obvious to us even though they are just as important (e.g. having a happy home-life). In addition, sometimes we can think of a goal (e.g. owning a dog) but after thinking about it realise that it is only important because it is helping us achieve something else that we really value (e.g. having company).

A lot of research shows that people who set and work towards specific goals are more confident and able to make changes to their lives—they are also more likely to achieve their goals and live life in a way they would like. However, a first step is realising what your goals really are, and if asthma makes it difficult to achieve them.

These questions are designed to help you think about things that matter to you in day-to-day life and how asthma affects them. We would like you to think about a range of different aspects of your life. You can use the list below as a guide:

| Personal: | looking after yourself, how you feel, things you enjoy |
| --- | --- |
| Home/Family: | relationships, duties, chores |
| Social: | friends, colleagues, community |
| Work: | employment, education, professional issues, career |
| Lifestyle: | diet, exercise, sleep, mental wellbeing, smoking, weight |

These questions are divided into three parts.

**Part 1** asks you to think about what’s important to you and you would like to achieve

**Part 2** asks you to think about why it is important to you

**Part 3** asks you to think about what you would like to do to manage your asthma better

Please take some time to complete these questions and take the completed booklet with you to your next asthma review. Your health professional will discuss these with you and help you to make specific plans to achieve your goals. This will help your health professional to tailor the care to your personal needs.

**1) Your goals in day-to-day life**

Write down what you would really like to do or achieve, even if you think it may not be related to your asthma. Think about things that you would like to do in your personal, home, work, and social life—things that you *need* to do, *want* to do and /or *enjoy* *doing*.

Then list them in order of priority— starting with 1 for the goal that matters to you most and that you would like to focus on at the moment. Finally, tell us whether you think your asthma makes it difficult for you to achieve each of these goals. To help you, we have put a completed example below.

| What do you really want to achieve?  (your goals) | Order of Priority | Does Asthma make it difficult to achieve? |
| --- | --- | --- |
|  |  |  |
| Example |  |  |
| - *I want to be able to work* | *1* | *Yes* |
| - *I want to lose weight* |  | *No* |
| - *I want to be able to sleep well* | *2* | *Yes* |
| - *I want to get back to being an active mum/dad* |  | *Not sure* |
| - *I want to be able to go out with friends* | *3* | *Yes* |

**2) Why is this important to you?**

Sometimes, a goal we come up with is an end in itself, which means that we want to achieve it for its own sake. But other times, a goal that quickly comes to mind is only important because it will help us to achieve something else. Finding out what we are really trying to achieve is crucial, as there may be more than one way of achieving it.

We now ask you to focus on your goals from section 1 and to think about *why* they are important to you. Below is an example of how to find out if a goal you have come up with is important for its own sake or it is important because it will help you achieve something else. Asking yourself ‘how would achieving this benefit me’ will help you identify what you really want to achieve. We ask you to do this exercise with **up to three** of your goals.

My children are old enough now not to need me there all the time and the extra money would make a big difference to our lives.

I would welcome the social contact with work colleagues

How would achieving this benefit me?

**Example Goal 1**

**I want to be able to work**

How would achieving this benefit me?

**Your Goal 1**

**Your Goal 2**

**Your Goal 3**

How would achieving this benefit me?

How would achieving this benefit me?

Having looked at your goals, we would now like you to think about **how you manage your asthma.**

**3) Your asthma management**

Asthma affects different people in different ways. Sometimes, people manage their asthma well and live life to the full. Other times, people have difficulties in managing their asthma and achieving what they want. Better management of asthma is likely to help you achieve the goals you have identified.

Below, we ask you to think about what is important to you regarding your asthma management. Write down up to 3 things that you would ideally do to manage your asthma better. This will help you realise how the way you manage your asthma is related to what you really want to achieve in day-to-day life. Tell us how important these things are to you and how confident you are that you can achieve them.

To help you, we have put a completed example below.

| Ideally, what do you wish you could do to manage your asthma better? | How important is this to you?  1= Not at all  5 = Extremely | How confident are you that you can achieve this?  1= Not at all  5 = Extremely |
| --- | --- | --- |
|  | 1 2 3 4 5      1 2 3 4 5      1 2 3 4 5 | 1 2 3 4 5      1 2 3 4 5      1 2 3 4 5 |
| Example | | |
| • *I want to keep asthma medication to minimum*  • *I want to avoid asthma triggers*  • *I want to keep symptom free* | 1  2 3 4 5    1 2 3 4 5    1 2 3 4 5 | 1  2 3 4 5    1  2 3 4 5    1 2 3 4 5 |
