# Supplementary material for: Goal-setting intervention in patients with active asthma: protocol for a pilot cluster-randomised controlled trial
Source: Trials. 2013 Sep 11;14:289. doi: 10.1186/1745-6215-14-289 (PMC3846716; doi:10.1186/1745-6215-14-289)
Supplement: Additional file 2 — Goal action plan. [file 1745-6215-14-289-S2.docx]

**Additional file 2: GOAL Action Plan**
