# Supplementary material for: Goal-setting intervention in patients with active asthma: protocol for a pilot cluster-randomised controlled trial
Source: Trials. 2013 Sep 11;14:289. doi: 10.1186/1745-6215-14-289 (PMC3846716; doi:10.1186/1745-6215-14-289)
Supplement: Additional file 3 — Practice nurse training schedule. [file 1745-6215-14-289-S3.docx]

**Additional file 3: Practice Nurse Training Schedule**

**Intervention & Control Groups**

- 12.15-13.00: Lunch
- 13.00-13.15: Introduction and Overview of the Trial
- 13.15-13.40: The Trial Protocol & Folder
- 13.40-14.00: The Review Consultation
- 14.00-14.15: Question & Answer session

**Intervention Group Only**

- 14.30-14.45: Introducing the Goal Setting Tool
  - - Aim and Purpose
    - Process and Application
    - Questions and Answers
- 14.45-16.00: Using the tool
  - - Observe 2 x 10 min video recorded consultations of using the goal tool with 15 minutes for discussion around issues/concerns
    - Completion of GOAL action plan by each nurse – discussion and address any issues.
- 16.00-16.15: Coffee
- 16.15-16.45: Question and Answer session
